# Genotyping of foot‐and‐mouth disease viruses collected in Sudan between 2009 and 2018

**DOI:** 10.1111/tbed.14472

**Published:** 2022-02-23

**Authors:** Yazeed A. Raouf, Jemma Wadsworth, Abdelghani Bin‐Tarif, Ashley R. Gray, Mohammed Habiela, Ameera A. Almutalb, Hanan Yousif, Maysa Ragab, Wefag Alfouz, Nussiba H. Ahmed, Inas Ibrahim, Ahmed M. Hassan, Markos Tibbo, Ahmad M. Almajali, Cornelis van Maanen, Nicholas A. Lyons, Donald P. King, Nick J. Knowles

**Affiliations:** ^1^ Foot‐and‐Mouth‐Disease Department Central Veterinary Research Laboratory (CVRL), Soba, Al Amarat Khartoum Sudan; ^2^ FAO World Reference Laboratory for FMD (WRLFMD) The Pirbright Institute, Woking Surrey UK; ^3^ Food and Agriculture Organization of the United Nations (FAO) Subregional Office for the Gulf Cooperation Council States and Yemen Abu Dhabi United Arab Emirates; ^4^ Faculty of Veterinary Medicine Department of Veterinary Clinical Sciences Jordan University of Science and Technology Irbid Jordan; ^5^ The European Commission for the Control of Foot‐and‐Mouth Disease (EuFMD) Food and Agriculture Organization of the United Nations (FAO) Rome Italy

**Keywords:** *Aphthovirus*, epidemiology, foot‐and‐mouth disease, nucleotide sequence, Sudan

## Abstract

Foot‐and‐mouth disease (FMD) is widely distributed in Sudan where outbreaks occur on an annual basis especially during the winter months (December‐February). This study aimed to increase our understanding of the epidemiological patterns of FMD in Sudan and connections to neighbouring countries by characterizing the genetic sequences of FMD viruses (FMDV) collected from samples collected in 10 Sudanese states over a 10‐year period (between 2009 and 2018). FMDV was detected in 91 of the 265 samples using an antigen‐detection ELISA. Three serotypes were detected: O (46.2%), A (34.0%), and SAT 2 (19.8%). Fifty‐two of these samples were submitted for sequence analyses, generating sequences that were characterized as belonging to O/EA‐3 (*n* = 17), A/AFRICA/G‐IV (*n* = 23) and SAT 2/VII/Alx‐12 (*n* = 12) viral lineages. Phylogenetic analyses provided evidence that FMDV lineages were maintained within Sudan, and also highlighted epidemiological connections to FMD outbreaks reported in neighbouring countries in East and North Africa (such as Ethiopia and Egypt). This study motivates continued FMD surveillance in Sudan to monitor the circulating viral lineages and broader initiatives to improve our understanding of the epidemiological risks in the region.

## 1 INTRODUCTION

Foot‐and‐mouth disease (FMD) remains a worldwide major constraint to animal production and international trade. The causative agent, FMD virus (FMDV; genus *Aphthovirus*, family *Picornaviridae*), exists as seven immunologically distinct serotypes (O, A, C, Asia 1, SAT 1, SAT 2 and SAT 3) and the virus is known to infect up to 70 cloven‐hoofed animal species (Grubman & Baxt, 2004). The epidemiology of FMD in Africa is complex. This is partly because the continent is resident to three out of the seven endemic FMDV pools (Pools 4, 5 and 6; Paton et al., 2009), and six out of the seven FMDV serotypes have been recorded (Rweyemamu et al., 2008), although serotype C has not been detected since 2004 (Paton et al., 2021). Sudan is one of the largest African countries (nearly 1.9 million km^2^) which has a resident population of more than 100 million FMD‐susceptible animals. It has 30–40 million cattle, 70 million small ruminants as well as a diverse range of FMD susceptible wildlife species that are located mostly in the Southern areas or in the Dinder National Park in Eastern Sudan (Anon, 2009). Sudan is recognized as an important crossroads between sub‐Saharan and Northern Africa, and between East and West Africa.

FMD has been known to be present in Sudan since 1903 (Abu Elzein, 1983) and previous studies have highlighted the contribution of FMD circulation in the country to the wider epidemiology of the disease in Africa (Bronsvoort et al., 2004; Hall et al., 2013; Rweyemamu et al., 2008; Ularamu et al., 2017). In sub‐Saharan African countries, FMD is endemic but clinical signs are often mild or inapparent particularly in sheep and goats and outbreaks are inconsistently reported to veterinary services. Together with the costs and logistics associated with the shipment of suspected FMDV samples, these issues result in infrequent submissions to international reference centres [such as the World Reference Laboratory for FMD (WRLFMD), Pirbright, United Kingdom] and consequent potential for biases in epidemiological inferences (Bronsvoort et al., 2004). To address these sampling and reporting issues, the Government of Sudan together with the Food and Agriculture Organization of the United Nations (FAO) and the European Commission for the Control of FMD (EuFMD) joined efforts in a Technical Corporation Programme (TCP/SUD/3401) to promote active surveillance of FMD in Sudan which was undertaken between 2012 and 2014. In this project extensive serosurveillance to detect antibodies against FMDV in cattle and small ruminants was carried out and the data has been reported elsewhere (Raouf et al., 2016, 2017). Furthermore, epithelium samples were collected during 2009–2018 from suspected disease events in 10 Sudanese states for virus detection, serotyping and molecular characterization. Four different FMDV serotypes have been reported to circulate in Sudan (O, A, SAT 1 and SAT 2) with SAT 1 last detected in 1976 (Habiela et al., 2010a). Vaccination against FMD has not been practiced to any appreciable extent in Sudan up to 2016 when vaccination of cattle in the Northern state was carried out but discontinued thereafter. In previous reports, type O has been detected more frequently while serotypes SAT 2 and A are found more sporadically (Abu Elzein, 1983; Abu Elzein et al., 1987; Habiela et al., 2010b; Raouf et al., 2016). The aim of this current study is to improve our understanding of the epidemiological patterns of FMD in Sudan and connections to neighbouring countries through characterizing FMDV in clinical samples.

## 2 MATERIALS AND METHODS

### 2.1 Clinical samples

During passive surveillance undertaken in the period 2009–2018, epithelium tissues (*n* = 265) were collected from suspected cases of FMD in cattle in 10 Sudanese states: Khartoum, River Nile, Gezira, Al Qadarif, White Nile, Blue Nile, North Kordofan, Kassala, North and South Darfur. Each year between 15 and 30 samples were collected except for 2013 and 2014 when more than 100 samples were collected. Sampled animals (all cattle) were mainly of around 1 to 5 years old, mostly female and occasionally male calves or bulls. Epithelium tissues were usually collected from recent cases in transport media comprised of equal amounts of glycerol and 0.04 M phosphate buffer, 0.001% phenol red, antibiotics and antimycotics (pH 7.2–7.6). Samples were kept refrigerated until reaching the laboratory where they were kept at −20°C.

### 2.2 FMDV detection and serotyping

All samples were screened for the presence of FMDV antigen at the Central Veterinary Research Laboratory (CVRL) either using the indirect sandwich ELISA kit (WRLFMD; Roeder & Le Blanc Smith, 1987) or using the monoclonal antibody based antigen detection and serotyping ELISA kit (IZSLER Biotech, Brescia, Italy; Grazioli et al., 2012; Grazioli et al., 2020). For sample testing, a suitable amount of epithelium was used to prepare a 10% suspension (W/V) using Glasgow minimum essential medium (GMEM), sterile sand and a pestle and mortar. Unprocessed epithelium was returned to −20°C deep freezer until shipment to the WRLFMD. The indirect sandwich ELISA kit employed polyclonal antibodies as trapping (rabbit antisera) and detecting (guinea pig antisera) antibodies in addition to antigen in each set of reagents while the IZSLER kit employed selected combinations of anti‐FMDV monoclonal antibodies (MAbs) as coated and conjugated antibodies. All ELISA reagents were prepared and supplied by the WRLFMD (the indirect sandwich ELISA kit) or by the Istituto Zooprofilattico Sperimentale della Lombardia e dell'Emilia Romagna (IZSLER kit) and were used according to the supplied protocols.

A subset of these FMDV positive samples (*n* = 52) were submitted to the WRLFMD (at the Pirbright Institute, Surrey, UK) for further study. Unprocessed epithelium samples in the described transport medium were dispatched, under dry ice, as dangerous biological substance category B UN 3373. At the WRLFMD, samples were tested by real time RT‐PCR (Callahan et al., 2002; Shaw et al., 2007) and virus isolation using primary bovine thyroid and IB‐RS‐2 cells as previously described (de Castro, 1964; Snowdon, 1966). Samples were considered FMDV negative if no CPE was observed for 48 h following blind passage of the first cell cultures after 48 h incubation at 37°C. Samples in which FMDV was identified (*n* = 91) are listed in Table 1 together with associated metadata; those lacking a WRLFMD reference number (*n* = 38) were not submitted to the WRLFMD.

### 2.3 Sequencing and phylogenetic analysis

VP1 sequences were generated using previously described methods (Knowles et al., 2016). Briefly, for each serotype two independent RT‐PCR assays were performed using the following primer pairs: O‐1C244F/EUR‐2B52R and O‐1C272F/EUR‐2B52R (type O); A‐1C562F/EUR‐2B52R and A‐1C612F/EUR‐2B52R (type A); and SAT2‐1C445F/SAT‐2B208R and SAT2‐P1‐1223F/SAT‐2B208R (type SAT 2). Sanger sequencing was performed on an ABI 3730xl DNA Analyzer (ABI Biosystems, Waltham, Massachusetts). Complete VP1 nucleotide sequences were aligned using BioEdit 7.0.5.3 (Hall, 1999) and Clustal W 1.83 (Thompson et al., 1994). Optimal nucleotide substitution models were computed for each serotype using MEGA 7 (Kumar et al., 2016). The maximum likelihood algorithm was used to construct phylogenetic trees using MEGA 7. One thousand bootstrap pseudo‐replicates were used to assess branching confidence.

## 3 RESULTS

### 3.1 FMD positive samples

All the 265 samples collected during this study (2009–2018) were of cattle origin. Approximately one‐third of these samples (91/265; 34.3%) were successfully serotyped using antigen detection ELISA. Fifty‐two of these samples were shipped to the WRLFMD and subjected to further testing. All 52 samples showed CPE within 24–48 h of being inoculated onto primary BTy cells and were subjected to VP1 nucleotide sequencing. Most of the typed samples were collected during the winter months (62/91; 68.1%): comprising 27/91 (29.7%) in December, 14/91 in January (15.4%), and 20/91 in February (22.0%). This compares to lower numbers of FMDV positive samples collected in November (1/91; 1.1%), March (17/91; 18.7%) and April (6/91; 6.6%). Only 6.6% (6/91) of the typed samples were collected between May and October.

**FIGURE 1:**
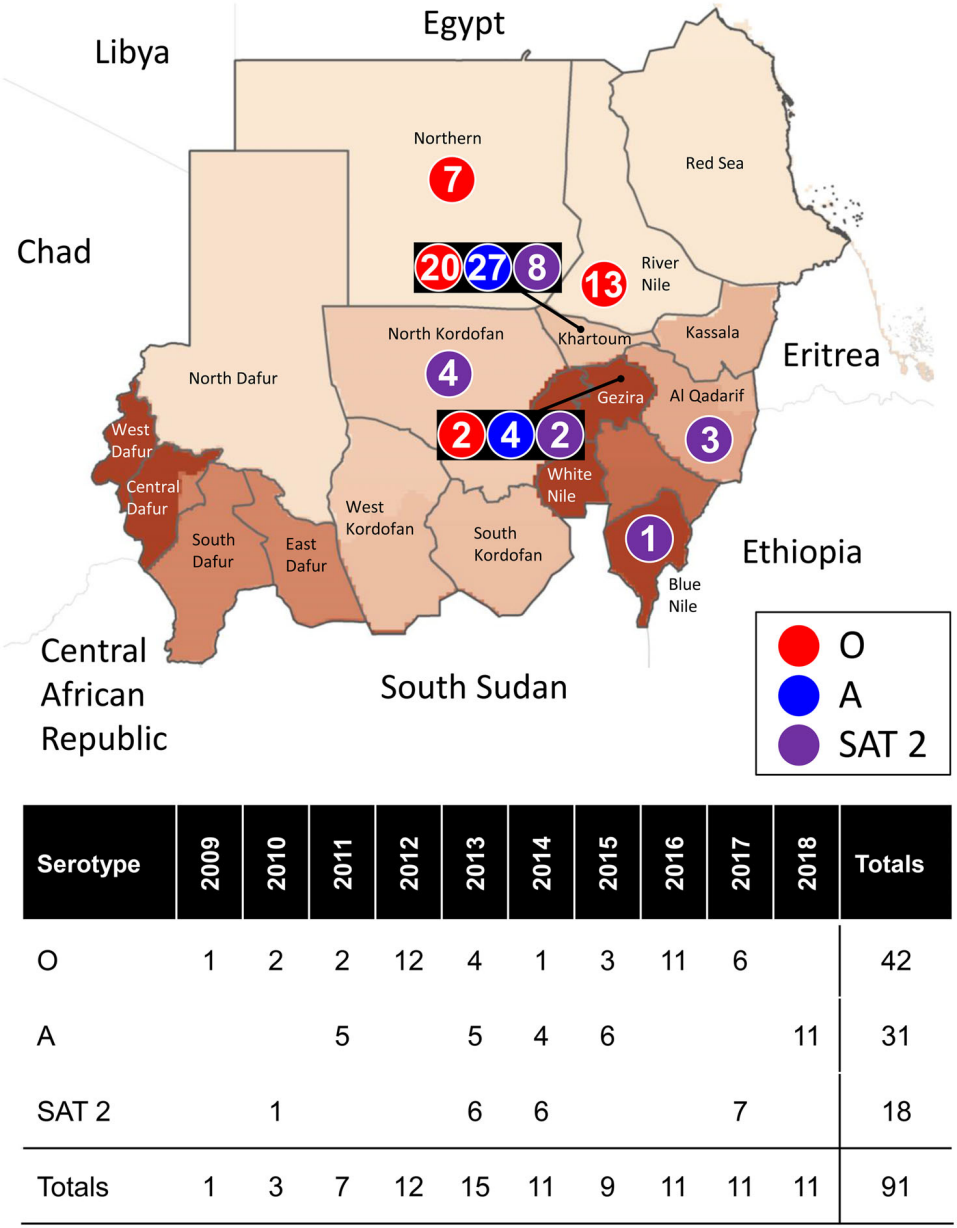
Geographical distribution of FMDV outbreaks in Sudan 2009–2018. Map represents Sudanese provinces where the intensity of the colour reflects on cattle density (FAO). Circles define numbers of samples that were serotyped using antigen detection ELISA (Red: serotype O; blue: serotype A; purple: serotype SAT 2)

The geographical distribution of FMDV positive samples collected in Sudan between 2009 and 2018 is described in Table 1 and Figure 1. FMDV was detected in 91 samples which were also serotyped. Disease events comprised three serotypes: O (42/91; 46.2%), SAT 2 (18/91; 19.8%) and A (31/91; 34.0%). FMD type O disease events extended over almost the entire reported period (Figure 1), apart from 2018, while serotype A was detected in 5 years (2011, 2013, 2014, 2015 and 2018) and serotype SAT 2 was detected in 4 years (2010, 2013, 2014 and 2017). Serotype A was detected in the Central States of Khartoum and Gezira. In comparison, serotypes O and SAT 2 had a wider geographical distribution (Figure 1). Serotype O was detected along the Nile basin, from the White Nile State in the South to Khartoum and Gezira in Central Sudan and up to the Northern State in Northern Sudan. Serotype SAT 2 was detected in Central Sudan at Khartoum, Gezira and North Kordofan and in addition in one South Eastern State (Blue Nile) and one Eastern Border State (Al Qadarif).

**TABLE 1:** Designation and origin of samples collected in Sudan 2009 to 2018 combined with results of FMDV characterization

Disease season	WRLFMD Ref. No.	CVRL ID	Collection date	Location	Host	Serotype	Topotype	Lineage	Sequence accession no.	Sequence reference
2009–2010	SUD/1/2009	12/2009	13/12/2009	Redwan, Omdurman, Khartoum	Cattle	O	EA‐3		KX258033	Ularamu et al. (2017)
	SUD/1/2010	1/2010	11/01/2010	Hilat Kuku, Khartoum	Cattle	O	EA‐3		KX258034	Ularamu et al. (2017)
	SUD/2/2010	5/2010	12/01/2010	Keriab, Khartoum North, Khartoum	Cattle	O	EA‐3		KX258035	Ularamu et al. (2017)
	SUD/4/2010	13B/2010	09/02/2010	Sheikan, Sheikan, North Kordofan	Cattle	SAT 2	VII	AIx‐12	KF112968	Hall et al. (2013)
2010–2011	SUD/1/2011	1/2011	28/02/2011	Keriab, Khartoum North, Khartoum	Cattle	A	AFRICA	G‐IV	MK422575	This work
	SUD/6/2011	14/2011	01/03/2011	Hilat Kuku, Khartoum North, Khartoum	Cattle	A	AFRICA	G‐IV	MK422576	This work
	SUD/7/2011	18/2011	01/03/2011	Shigla, Khartoum North, Khartoum	Cattle	A	AFRICA	G‐IV	MK422577	This work
	SUD/12/2011	31/2011	03/03/2011	Sarha, Omdurman, Khartoum	Cattle	A	AFRICA	G‐IV	MK422579	This work
	SUD/13/2011	38/2011	03/03/2011	Moilah, Khartoum	Cattle	A	AFRICA	G‐IV	MK422578	This work
	SUD/9/2011	28/2011	03/03/2011	Moilah, Khartoum	Cattle	O	EA‐3		MK422556	This work
	SUD/11/2011	31/2011	03/03/2011	Moilah, Khartoum	Cattle	O	EA‐3		KX258036	Ularamu et al. (2017)
2011–2012	SUD/1/2012	Feb/2012‐R. Nile 2/5	01/02/2012	River Nile	Cattle	O	EA‐3		MK422557	This work
	SUD/2/2012	Feb/2012‐R. Nile 3/5	01/02/2012	River Nile	Cattle	O	EA‐3		MK422558	This work
	SUD/3/2012	Feb/2012‐R. Nile 2/7	01/02/2012	River Nile	Cattle	O	EA‐3		MK422559	This work
	SUD/5/2012	Feb/2012‐R. Nile 4/7	01/02/2012	River Nile	Cattle	O	EA‐3		MK422560	This work
	SUD/6/2012	Feb/2012‐R. Nile 2/11	01/02/2012	River Nile	Cattle	O	EA‐3		MK422561	This work
	None^b^	R. Nile 1/5	01/02/2012	River Nile	Cattle	O	nd^c^			
	None^b^	R. Nile 1/7	01/02/2012	River Nile	Cattle	O	nd			
	None^b^	Feb/2012‐R. Nile (55)^a^	13/02/2012	River Nile	Cattle	O	nd			
	None^b^	Feb/2012‐R. Nile (56) ^a^	13/02/2012	River Nile	Cattle	O	nd			
	None^b^	R. Nile (60)	19/03/2012	Alkoa, River Nile	Cattle	O	nd			
	None^b^	R. Nile (61)	19/03/2012	River Nile	Cattle	O	nd			
2012–2013	None^b^	R. Nile 66	10/12/2012	Shendi, River Nile	Cattle	O	nd			
	None^b^	Ep/1/2013	13/01/2013	Jabal Alawlia, Khartoum	Cattle	O	nd			
	None^b^	Ep. 5‐6/2013	21/01/2013	Al Kamleen, Gezira	Cattle	O	nd			
	SUD/1/2013	Feb/2013‐Kh 1/4	19/02/2013	Khartoum State	Cattle	A	AFRICA	G‐IV	MK422580	This work
	SUD/3/2013	April/2013‐Kh 8	16/04/2013	Soba, Khartoum	Cattle	SAT 2	VII	Alx‐12	MK422598	This work
2013–2014	SUD/4/2013	Gez 2	30/12/2013	Alzrieba, Gezira	Cattle	O	EA‐3		MK422562	This work
	SUD/5/2013	Kh 10	31/12/2013	Mahlab 2, Khartoum	Cattle	SAT 2	VII	Alx‐12	MK422599	This work
	SUD/9/2013	N. Kordfan 5	31/12/2013	Alhmadia, North Kordafan	Cattle	SAT 2	VII	Alx‐12	MK422600	This work
	SUD/10/2013	Gez 3	31/12/2013	Alktateib, Gezira	Cattle	A	AFRICA	G‐IV	MK422581	This work
	SUD/11/2013	Gez 5	31/12/2013	Alktateib, Gezira	Cattle	A	AFRICA	G‐IV	MK422582	This work
	SUD/12/2013	Gez 7	31/12/2013	Alktateib, Gezira	Cattle	A	AFRICA	G‐IV	MK422583	This work
	SUD/13/2013	Gez 9	31/12/2013	Alktateib, Gezira	Cattle	A	AFRICA	G‐IV	MK422584	This work
	SUD/4/2014	Ged 17	01/01/2014	Gedarif State	Cattle	SAT 2	VII	Alx‐12	MK422601	This work
	SUD/7/2014	B. Nile 11	01/01/2014	Blue Nile State	Cattle	SAT 2	VII	Alx‐12	MK422602	This work
	SUD/11/2014	Gez 15	01/01/2014	Gezira State	Cattle	SAT 2	VII	Alx‐12	MK422603	This work
	None^b^	Kh 5	31/12/2013	Mahlab 2, Khartoum	Cattle	SAT 2	nd			
	None^b^	Kh 6	31/12/2013	Mahlab 2, Khartoum	Cattle	O	nd			
	None^b^	N. Kordfan 3	31/12/2013	Alhmadia, North Kordafan	Cattle	SAT 2	nd			
	None^b^	N. Kordfan 6	31/12/2013	Alhmadia, North Kordafan	Cattle	SAT 2	nd			
	None^b^	R. Nile 2	Jan‐2014	River Nile	Cattle	O	nd			
	None^b^	Ged 10	01/01/2014	Al Qadarif State	Cattle	SAT 2	nd			
	None^b^	Ged 16	01/01/2014	Al Qadarif State	Cattle	SAT 2	nd			
	None^b^	Gez 13	01/01/2014	Gezira State	Cattle	SAT 2	nd			
2014–2015	SUD/18/2014	2/2015	24/12/2014	Al Tibna, Khartoum	Cattle	A	AFRICA	G‐IV	MK422585	This work
	SUD/20/2014	4/2015	24/12/2014	Al Tibna, Khartoum	Cattle	A	AFRICA	G‐IV	MK422586	This work
	SUD/21/2014	5/2015	24/12/2014	Al Tibna, Khartoum	Cattle	A	AFRICA	G‐IV	MK422587	This work
	SUD/22/2014	6/2015	24/12/2014	Al Tibna, Khartoum	Cattle	A	AFRICA	G‐IV	MK422588	This work
	None^b^	Ep‐1/2015	Apr‐15	Kuku, Khartoum	Cattle	A	nd			
	None^b^	Ep‐3/2015	Apr‐15	Kuku, Khartoum	Cattle	A	nd			
	None^b^	Ep‐6/2015	Apr‐15	Kuku, Khartoum	Cattle	O	nd			
	None^b^	Ep‐7/2015	Apr‐15	Kuku, Khartoum	Cattle	O	nd			
	None^b^	Ep‐9/2015	Apr‐15	Kuku, Khartoum	Cattle	O	nd			
2015–2016	None^b^	Ep‐10/2015	06/12/2015	Safola, Khartoum	Cattle	A	nd			
	None^b^	Ep‐11/2015	06/12/2015	Safola, Khartoum	Cattle	A	nd			
	None^b^	Ep‐12/2015	06/12/2015	Safola, Khartoum	Cattle	A	nd			
	None^b^	Ep‐13/2015	06/12/2015	Safola, Khartoum	Cattle	A	nd			
	None^b^	Ep‐3/2016	Sep‐16	Algadeada, Khartoum	Cattle	O	nd			
	None^b^	Ep4/2016	Sep‐16	Algadeada, Khartoum	Cattle	O	nd			
	None^b^	Ep‐5/2016	Sep‐16	Algadeada, Khartoum	Cattle	O	nd			
	None^b^	Ep‐6/2016	Sep‐16	Algadeada, Khartoum	Cattle	O	nd			
2016–2017	None^b^	Ep‐9/2016	Nov‐2016	Dongla, Northern state	Cattle	O	nd			
	None^b^	Ep‐/2017 (1) (1‐5)	25/12/2016	border control, Northern state	Cattle	O	nd			
	None^b^	Ep‐/2017 (2) (1‐5)	25/12/2016	border control, Northern state	Cattle	O	nd			
	None^b^	Ep‐/2017 (3) (1‐5)	25/12/2016	border control, Northern state	Cattle	O	nd			
	None^b^	Ep‐/2017 (4) (1‐5)	25/12/2016	border control, Northern state	Cattle	O	nd			
	None^b^	Ep‐/2017 (5) (1‐5)	25/12/2016	border control, Northern state	Cattle	O	nd			
	SUD/1/2016	1/2016	25/12/2016	border control, Northern state	Cattle	O	EA‐3		MK422563	This work
	SUD/2/2017	1/2017	04/01/2017	Mahlab 3, Kuku, Khartoum	Cattle	O	EA‐3		MK422564	This work
	SUD/3/2017	2/2017	04/01/2017	Mahlab 3, Kuku, Khartoum	Cattle	O	EA‐3		MK422565	This work
	SUD/4/2017	11/2017	09/01/2017	Al Rdwan, Khartoum	Cattle	O	EA‐3		MK422566	This work
	SUD/5/2017	3/2017	07/02/2017	Al Rdwan, Khartoum (No. 1)	Cattle	O	EA‐3		MK422567	This work
	SUD/6/2017	4/2017	07/02/2017	Al Rdwan, Khartoum (No. 2)	Cattle	SAT 2	VII	Alx‐12	MK422604	This work
	SUD/7/2017	5/2017	07/02/2017	Al Rdwan, Khartoum (No. 3)	Cattle	O	EA‐3		MK422568	This work
	SUD/7/2017	5/2017	07/02/2017	Al Rdwan, Khartoum (No. 3)	Cattle	SAT 2	VII	Alx‐12	MK422605	This work
	SUD/9/2017	7/2017	21/02/2017	Al Rdwan, Khartoum (No. 4)	Cattle	SAT 2	VII	Alx‐12	MK422606	This work
	SUD/12/2017	14/2017	21/02/2017	Al Rdwan, Khartoum (No. 2)	Cattle	SAT 2	VII	Alx‐12	MK422607	This work
	SUD/14/2017	8/2017	04/06/2017	Al Aelfon, Khartoum (No. 1)	Cattle	SAT 2	VII	Alx‐12	MK422608	This work
	SUD/15/2017	9/2017	04/06/2017	Al Aelfon, Khartoum (No. 2)	Cattle	O	EA‐3		MK422569	This work
2017–2018	SUD/3/2018	1/2018	06/02/2018	Al Shigla, Khartoum	Cattle	A	AFRICA	G‐IV	MK422589	This work
	SUD/4/2018	2/2018	07/02/2018	Al Shigla, Khartoum	Cattle	A	AFRICA	G‐IV	MK422590	This work
	SUD/6/2018	5/2018	20/03/2018	Mahlab 2, Kuku, Khartoum	Cattle	A	AFRICA	G‐IV	MK422591	This work
	SUD/7/2018	5‐a/2018	20/03/2018	Mahlab 2, Kuku, Khartoum	Cattle	A	AFRICA	G‐IV	MK422592	This work
	SUD/8/2018	6/2018	20/03/2018	Mahlab 2, Kuku, Khartoum	Cattle	A	AFRICA	G‐IV	MK422593	This work
	SUD/9/2018	9/2018	22/03/2018	Safola, Khartoum	Cattle	A	AFRICA	G‐IV	MK422594	This work
	SUD/10/2018	11/2018	28/03/2018	Al Shigla, Khartoum	Cattle	A	AFRICA	G‐IV	MK422595	This work
	SUD/11/2018	12/2018	28/03/2018	Al Shigla, Khartoum	Cattle	A	AFRICA	G‐IV	MK422596	This work
	SUD/12/2018	13/2018	28/03/2018	Al Shigla, Khartoum	Cattle	A	AFRICA	G‐IV	MK422597	This work
	None^b^	Ep‐4/2018	20/03/2018	Mahlab 2, Kuku, Khartoum	Cattle	A	nd			
	None^b^	Ep‐7/2018	20/03/2018	Mahlab 2, Kuku, Khartoum	Cattle	A	nd			

^a^ Identity number designated by the Department of Animal Health and Epizootic Diseases Control.

^b^ Sample not submitted to the WRLFMD.

^c^ Genotyping not done.

**FIGURE 2:**
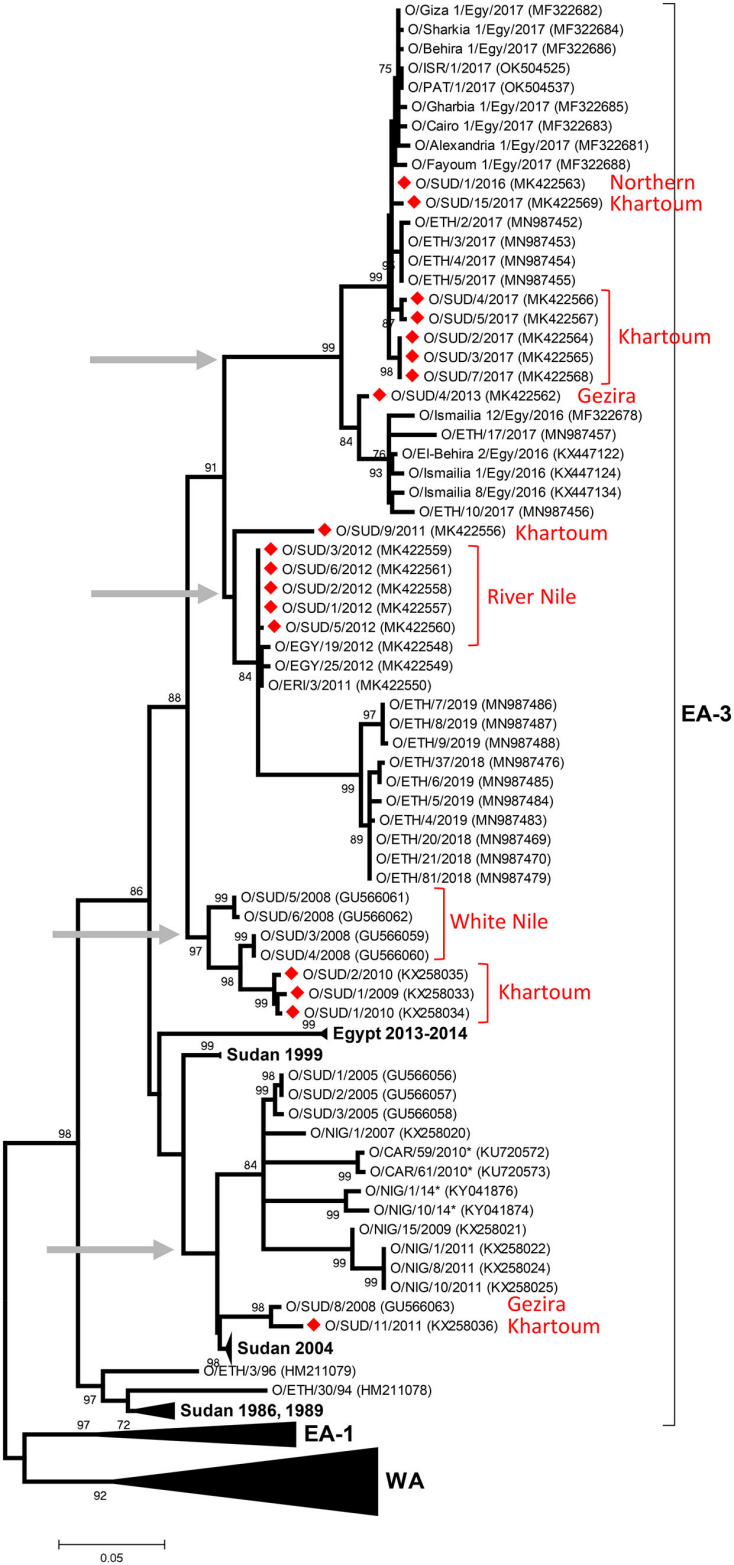
Midpoint‐rooted maximum likelihood tree showing the relationships between the VP1 sequences of the 2009–2017 serotype O viruses from Sudan (indicated with red diamonds) and other contemporary and reference viruses. Four distinct clades which contained Sudanese sequences are indicated by grey arrows. Bootstrap values of 70% and above are shown. ^*^Reference number not assigned by the WRLFMD

### 3.2 Phylogenetic analysis

All FMDV positive samples generated a single VP1 sequence apart from SUD/7/2017 where a mixture of O and SAT 2 were recovered. Antigen ELISA typing of the SUD/7/2017 BTy1 material showed it to be SAT 2, so sample contamination with FMDV type O cannot be excluded. The most appropriate nucleotide substitution models for each serotype were found to be the Tamura‐Nei (TN‐93) model, gamma distributed with invariant sites (G+I) (type O and type SAT 2); and the Hasegawa‐Kishino‐Yano (HKY) model, G+I (type A).

### 3.3 Serotype O

All the eighteen serotype O viruses that were genotyped during this study fell within the O/EA‐3 topotype (Figure 2). These sequences represent at least four distinct clades (indicated by grey arrows in Figure 2); three of which occurred during the same period (2009–2011), while a fourth cluster contained more recent isolates from 2013, 2016 and 2017. Nucleotide identities within clusters were mostly >95%, in contrast to sequence differences among clusters of between 5% and 7%. The first cluster contained isolate SUD/11/2011 from this study, together with older Sudanese FMDVs collected between 2004 and 2008, with Nigerian viruses collected between 2007 and 2014 and two Cameroon viruses collected in 2010. The second cluster contained three isolates: SUD/1/2009, SUD/1/2010 and SUD/2/2010 related to FMDV sequences from Sudan in 2008, while the third cluster contained five identical sequences for FMDV isolates collected in 2012, one collected a year earlier (O/SUD/9/2011) as well as samples from Eritrea (2011), Ethiopia (O/ETH/59/2011) and Egypt (2012). The final clade comprised seven Sudanese FMDV isolates collected in 2016/17 and an earlier sample from 2013 (O/SUD/4/2013). Viruses in this clade are part of a larger temporal phylogenetic cluster representative of O/EA‐3 outbreaks reported in Ethiopia, Egypt, Israel and Palestine in 2017.

### 3.4 Serotype A

**FIGURE 3:**
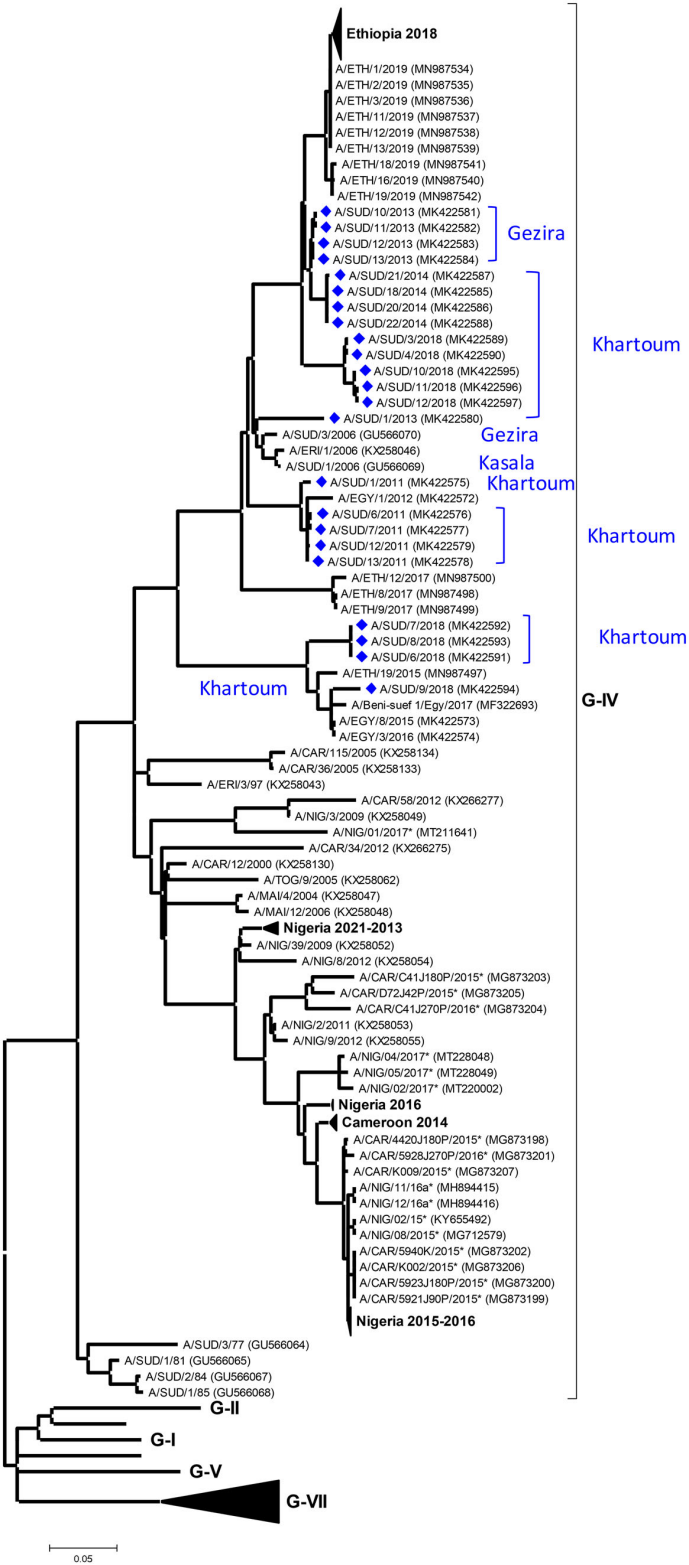
Midpoint‐rooted maximum likelihood tree showing the relationships between the VP1 sequences of the 2011–2018 serotype A viruses from Sudan (indicated with blue diamonds) and other contemporary and reference viruses. Bootstrap values of 70% and above are shown. ^*^Reference number not assigned by the WRLFMD

Twenty‐three serotype A viruses belonging to the A/AFRICA/G‐IV lineage were genotyped (Figure 3) during this study. Sudanese FMDVs, collected during 2018, belonged to two genetic clades (12.8–14.6% nt difference), with older ones represented in ancestral clusters. One of these contemporary clusters containing A/SUD/6‐9/2018 also contained FMDVs collected during 2015–2017 in Egypt and Ethiopia in 2015 (A/ETH/19/2015). At the common root of both clusters were sequences for FMDVs collected during 2006 from Sudan and Eritrea.

### 3.5 Serotype SAT 2

**FIGURE 4:**
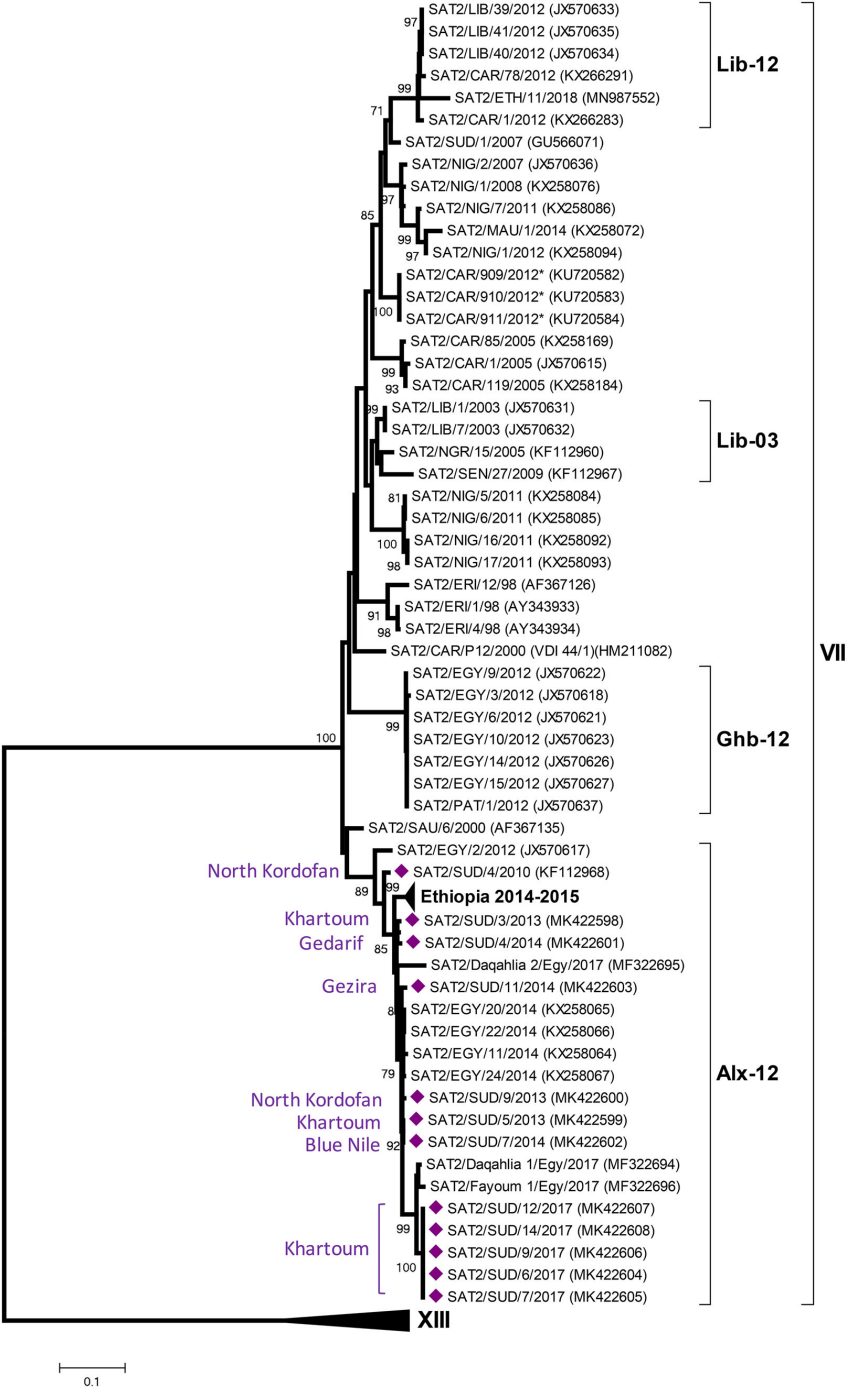
Midpoint‐rooted maximum likelihood tree showing the relationships between the VP1 sequences of the 2010–2017 serotype SAT 2 viruses from Sudan (indicated with purple diamonds) and other contemporary and reference viruses. Bootstrap values of 70% and above are shown. ^*^Reference number not assigned by the WRLFMD

Twelve serotype SAT 2 viruses within the SAT 2 topotype VII, distributed between 2010 and 2017 were characterized (Figure 4). All of these Sudanese viruses belonged to the Alx‐12 lineage which also contains sequences from FMDVs collected from other countries including Egypt 2012, 2014 and 2017. Older Sudanese viruses (from 1977 and 2007–2008) belonged to topotype XIII and were related to viruses from Ethiopia.

## 4 DISCUSSION

During the study period (2009–2018), clinical cases in cattle were observed year‐round over a wide geographical distribution with relatively more cases reported in the winter months (December–February). So far, four FMDV serotypes have been reported in Sudan: O, A, SAT 1 and SAT 2 (Abu Elzein, 1983), although no cases due to the SAT 1 serotype were detected in this study or have been recorded in Sudan since 1976 (Abu Elzein & Crowther, 1979). The low incidence of SAT1 in Sudan is supported by serological data for SAT1‐specific antibodies in comparison to serotypes O and A (Habiela et al., 2010a; Raouf et al., 2009). Serotype O was the most frequently detected; findings which are also supported by recent serological data (Raouf et al., 2016) showing higher prevalence of serotype O specific antibodies (60.2%) compared to serotype A (30.0%) and serotype SAT 2 (12.3%). Nucleotide distances of more than 15% (for serotypes O and A) or 20% (for serotypes SAT 1 and SAT 2) are used to classify isolates into different topotypes (Knowles & Samuel, 2003; Samuel & Knowles, 2001), while nucleotide differences of between 5% and 15% indicate distinct virus lineages (Bronsvoort et al., 2004). The Sudanese sequences reported here differed from the prototype strains of the relevant topotypes (Knowles et al., 2016) by 7.7–12.1% (O/SUD/2/86), 13.8–16% (O/ETH/3/2004), 15.2–17.8% (O/ETH/2/2006), 12.5–15.5% (O/ETH/1/2007), 13.4–16% (A/SUD/3/77), 7.4–9.9% (SAT2/SAU/6/2000) and 11.9–14.4% (SAT2/CAR/8/2005). These differences indicated the continued divergence of virus lineages within each topotype.

Serological findings from 2013 (Raouf et al., 2016) demonstrate that serotype O is present along the Nile basin up to Khartoum and in Western, Eastern and Northern Sudan. A previous study (Habiela et al., 2010b) highlighted that within‐country circulation is an important mechanism by which serotype O virus is maintained in Sudan. New FMDV sequence data generated here continues to demonstrate that serotype O is maintained in the country over successive years without much requirement for the introduction of viruses via transboundary pathways. This point is supported by the presence of at least two viral lineages within the phylogenetic tree that circulated during the same time period (2009–2011) and were related phylogenetically (nt. id. ≥95%) to earlier viral sequences from Sudan (2004–2011 and 2008–2010). The two clades containing Sudanese and Egyptian sequences are indicative of the northerly spread of FMDV O/EA‐3 from Sudan, which based on the dates occurred as separate independent events during 2011–2012, 2013–2016 and 2016–2017. One of these clades also contains viruses from Eritrea and Ethiopia, highlighting other potential transboundary transmission pathways to and from other countries in the neighbourhood.

Data for serotype A sequence also support the concept that FMDV lineages are maintained solely within Sudan. The phylogenetic tree contains two lineages that comprise sequences only from Sudan collected from different years (2013–2014 and 2018). In common with the serotype O data, there was also evidence for the northerly spread of A/AFRICA/G‐IV into Egypt (during 2011–2012). Epidemiological links to FMD cases in Ethiopia are highlighted in a second clade that contained Egyptian sequences (from 2015 to 2017) and more recent sequences collected from Sudan (from 2018), where the Ethiopian sequence (A/ETH/19/2015) pre‐dated those for the Sudanese samples. More broadly, the serotype A (and serotype O) phylogenetic trees highlight the relationship between recent sequences for FMDVs collected in Ethiopia (Gizaw et al., 2020) and contemporary data from Sudan. For both serotypes, there are examples where Sudanese sequences have a more basal ancestral location in the tree, for example, the relationships between O/Sudan/2012 and O/Ethiopia/2018–2019 and A/Sudan/2013–2014 and A/Ethiopia/2018–2019, respectively. These results suggest that these FMDVs could have spread from Sudan into Ethiopia; however, this interpretation of the data should be treated cautiously in view of the likely high number of unsampled FMD cases in both countries where the sampling density is very low and susceptible livestock populations are high.

Serotype SAT 2 was the last FMDV serotype to be detected in Sudan in 1976 (Abu Elzein & Crowther, 1979) and 18 Sudanese isolates of serotype SAT 2 were detected during four of the years during this study. All of these sequences from 2010 to 2017 were characterized as belonging to the SAT 2/VII/Alx‐12 lineage that has spread widely in the region to cause FMD outbreaks in countries such as Egypt, Palestine and Israel. In common with serotypes O and A, sequences for viruses from this lineage collected from Sudan are earlier compared to those for viruses sampled in Ethiopia. The SAT 2/VII/Alx‐12 lineage is distinct to earlier viral clades detected in Sudan such as those that have spread previously from East Africa into West Africa (Habiela et al., 2010b; Ularamu et al., 2017: highlighted by presence of SAT2/SUD/1/2007 in the phylogenetic tree).

No previous reports have described clinical FMD in Sudan in species other than cattle (Abu Elzein, 1983; Habiela et al., 2010a; Raouf et al., 2010). Recent serological studies suggested a relatively low seroprevalence of non‐structural protein antibodies among small ruminants at around 14%, except in Khartoum (Habiela et al., 2009) and Blue Nile State (Raouf, 2015; Raouf et al., 2017). This may indicate a varied role of sheep in the epidemiology of FMD. FMD infection in wild ruminants in Sudan has not been investigated.

In summary, FMD infection in Sudan remains regionally significant and this study highlights the epidemiological connections between FMDV sequences collected in Sudan and neighbouring countries such as Ethiopia and Egypt. Due to incomplete and convenience‐biased sampling, it should be recognized that there are limitations in our understanding of the transboundary connectivity in the region and the sequences reported here provide only a crude snapshot survey of the underlying FMD transmission events. Recognition of border areas as particularly risky hotspots for the introduction of FMDVs is of high importance in developing a risk‐based control strategy especially when resources are limited. In this context, continued studies are warranted to improve our understanding of FMD epidemiology in Sudan and risk‐pathways in the region.

## CONFLICT OF INTEREST

The authors declare no commercial or financial conflict of interest.

## ETHICAL STATEMENT

The authors confirm that the ethical policies of the journal, as noted on the journal's author guidelines page, have been adhered to. No ethical approval was required as this this study did not involve any experimental animal protocols.

## Data Availability

All nucleotide sequences generated were submitted to GenBank and accession numbers can be found in Table 1.

## References

[tbed14472-bib-0001] Abu Elzein, E. M. E. (1983). Foot and mouth disease in the Sudan. Revue Scientifique et Technique de l'Office International des Epizooties, 2, 177–188. 10.20506/rst.2.1.106 32993228

[tbed14472-bib-0002] Abu Elzein, E. M. E. , & Crowther, J. R. (1979). Serological comparison of a type SAT2 foot‐and‐mouth disease virus isolate from Sudan with other type SAT2 strains. Bulletin of Animal Health and Production in Africa, 27, 245–248. 232850

[tbed14472-bib-0003] Abu Elzein, E. M. E. , Newman, B. J. , Crowther, J. R. , Barnett, I. T. R. , & McGrane, J. J. (1987). The prevalence of antibodies against foot‐and‐mouth disease in various species of Sudanese livestock following natural infection. Revue d’élevage et médecine vétérinaire des pays tropicaux, 40, 7–12. 2832908

[tbed14472-bib-0004] Anonymous (2009). Sudan's fourth national report to the convention on biological diversity (p. 77). The Higher Council for Environment and Natural Resources (HCENR), Ministry of Environment and Physical Development, Government of the Republic of Sudan. https://www.cbd.int/doc/world/sd/sd‐nr‐04‐en.pdf

[tbed14472-bib-0005] Bronsvoort, B. M. de, C. , Radford, A. D. , Tanya, I. V. N. , Nfon, C. , Kitching, R. P. , & Morgan, K. L. (2004). Molecular epidemiology of foot‐and‐mouth disease viruses in the Adamawa Province of Cameroon. Journal of Clinical Microbiology, 42, 2186–2196. 10.1128/JCM.42.5.2186-2196.2004 15131187 PMC404612

[tbed14472-bib-0006] Callahan, J. D. , Brown, F. , Osorio, F. A. , Sur, J. H. , Kramer, E. , Long, G. W. , Lubroth, J. , Ellis, S. J. , Shoulars, K. S. , Gaffney, K. L. , Rock, D. L. , & Nelson, W. M. (2002). Use of a portable real‐time reverse transcriptase‐polymerase chain reaction assay for rapid detection of foot‐and‐mouth disease virus. Journal of the American Veterinary Medical Association, 220, 1636–1642. 10.2460/javma.2002.220.1636 12051502

[tbed14472-bib-0007] de Castro, M. P. (1964). Behavior of foot‐and‐mouth disease virus in cell culture: Susceptibility of the IB‐RS‐2 swine cell line. Arquivos do Instituto Biologico Sao Paulo, 31, 63–78.

[tbed14472-bib-0008] Gizaw, D. , Tesfaye, Y. , Wood, B. A. , Di Nardo, A. , Shegu, D. , Muluneh, A. , Bilata, T. , Belayneh, R. , Fentie, A. , Asgdome, H. , Sombo, M. , Rufael, T. , Tadesse Woldemariyam, F. , Khan, F. , Yami, M. , Gelaye, E. , Wadsworth, J. , Knowles, N. J. , & King, D. P. (2020). Molecular characterization of foot‐and‐mouth disease viruses circulating in Ethiopia between 2008 and 2019. Transboundary and Emerging Diseases, 67, 2983–2992. 10.1111/tbed.13675 32574400

[tbed14472-bib-0009] Grazioli, S. , Ferris, N. , Dho, G. , Spagnoli, E. , & Brocchi, E. (2012). Ready‐to‐use ELISA kit for FMDV diagnosis and serotyping tailored for Africa. Session of the Research Group of the Standing Technical Committee of EuFMD, Jerez, Spain, 29 Oct‐31 Oct 2012.

[tbed14472-bib-0010] Grazioli, S. , Ferris, N. P. , Dho, G. , Pezzoni, G. , Morris, A. S. , Mioulet, V. , & Brocchi, E. (2020). Development and validation of a simplified serotyping ELISA based on monoclonal antibodies for the diagnosis of foot‐and‐mouth disease virus serotypes O, A, C and Asia 1. Transboundary and Emerging Diseases, 67, 3005–3015. 10.1111/tbed.13677 32530134

[tbed14472-bib-0011] Grubman, M. J. , & Baxt, B. (2004). Foot‐and‐mouth disease. Clinical Microbiology Reviews, 17, 465–493. 10.1128/CMR.17.2.465-493.2004 15084510 PMC387408

[tbed14472-bib-0012] Habiela, M. , Raouf, Y. A. , & Nur Eldin, H. , (2009). Sero‐survey of anti‐foot and mouth disease virus antibodies in sheep and goats in Khartoum state, Sudan. The Sudan Journal of Veterinary Research, 24, 61–64.

[tbed14472-bib-0013] Habiela, M. , Alamin, M. A. G. , Raouf, Y. A. , & Ali, Y. A. (2010a). Epizootiological study of foot and mouth disease in the Sudan: The situation after two decades. Veterinarski Arhiv, 80, 11–26.

[tbed14472-bib-0014] Habiela, M. , N. P. Ferris , G. H. Hutchings , J. Wadsworth , S. M. Reid , M. Madi , K. Ebert , K. J. Sumption , N. J. Knowles , D. P. King , & D. J. Paton , (2010b). Molecular characterization of foot‐and‐mouth disease viruses collected from Sudan. Transboundary and Emerging Diseases, 57, 305–314. 10.1111/j.1865-1682.2010.01151.x 20626708

[tbed14472-bib-0015] Hall, T. A. (1999). BioEdit: A user‐friendly biological sequence alignment editor and analysis program for Windows 95/98/NT. Nucleic Acids Symposium Series, 41, 95–98.

[tbed14472-bib-0016] Hall, M. D. , Knowles, N. J. , Wadsworth, J. , Rambaut, A. , & Woolhouse, M. E. (2013). Reconstructing geographical movements and host species transitions of foot‐and‐mouth disease virus serotype SAT 2. mBio, 4, e00591‐13. 10.1128/mBio.00591-13 PMC3812709 24149511

[tbed14472-bib-0017] Knowles, N. J. , & Samuel, A. R. (2003). Molecular epidemiology of foot‐and‐mouth disease virus. Virus Research, 91, 65–80. 10.1016/s0168-1702(02)00260-5 12527438

[tbed14472-bib-0018] Knowles, N. J. , Wadsworth, J. , Bachanek‐Bankowska, K. , & King, D. P. (2016). VP1 sequencing protocol for foot and mouth disease virus molecular epidemiology. Revue Scientifique et Technique de l'Office International des Epizooties, 35, 741–755. 10.20506/rst.35.3.2565 28332654

[tbed14472-bib-0019] Kumar, S. , Stecher, G. , & Tamura, K. (2016). MEGA7: Molecular evolutionary genetics analysis version 7.0 for bigger datasets. Molecular Biology and Evolution, 33, 1870–1874. 10.1093/molbev/msw054 27004904 PMC8210823

[tbed14472-bib-0020] Paton, D. J. , Sumption, K. J. , & Charleston, B. (2009). Options for control of foot‐and‐mouth disease: Knowledge, capability and policy. Philosophical Transactions of the Royal Society B: Biological Sciences, 364, 2657–2667. 10.1098/rstb.2009.0100 PMC2865093 19687036

[tbed14472-bib-0021] Paton, D. J. , Di Nardo, A. , Knowles, N. J. , Wadsworth, J. , Pituco, E. M. , Cosivi, O. , Rivera, A. M. , Bakkali Kassimi, L. , Brocchi, E. , de Clercq, K. , Carrillo, C. , Maree, F. F. , Singh, R. K. , Vosloo, W. , Park, M.‐K. , Sumption, K. J. , Ludi, A. B. , & King, D. P. (2021). The history of foot‐and‐mouth disease virus serotype C: The first known extinct serotype? Virus Evolution, 7, 2057‐1577. 10.1093/ve/veab009 PMC8102019 35186323

[tbed14472-bib-0022] Raouf, Y. A. (2015). Some observations on the comparative performance of two 3‐ABC ELISAs in an area of endemicity. Bulletin of Animal Health and Production in Africa, 63, 129–138.

[tbed14472-bib-0023] Raouf, Y. A. , Ali, B. H. , Khair, S. M. , & Amin, A. M. (2009). The Prevalence of antibodies against SAT1 foot‐and‐mouth disease virus in cattle in Khartoum State: Epidemiological significance. Bulletin of Animal Health and Production in Africa, 57, 339–347. 10.4314/bahpa.v57i4.51680

[tbed14472-bib-0024] Raouf, Y. A. , Ali, B. , Amin, M. A. I. , & Al Shallali, A. M. (2010). Laboratory investigation of three outbreaks of foot‐and‐mouth disease at central Sudan and the disease type situation. Bulletin of Animal Health and Production in Africa, 58, 308–314.

[tbed14472-bib-0025] Raouf, Y. A. , Tamador, M. A. A. , Nahid, A. I. , & Shaza, M. (2012). A survey for antibodies against current infection of foot‐and‐mouth disease virus in Sudanese cattle, sheep and goats using neutralization test. Bulletin of Animal Health and Production in Africa, 60, 351–358.

[tbed14472-bib-0026] Raouf, Y. A. , Yousif, H. , Almutlab, A. A. , Hassan, A. A. , Ibra, A. , Tibbo, M. , & Al‐Majali, A. (2016). Sero‐epidemiology of foot‐and‐mouth disease in Sudan. Bulletin of Animal Health and Production in Africa, 64, 443–451.

[tbed14472-bib-0027] Raouf, Y. A. , Yousif, H. , Almutlab, A. A. , Hassan, A. A. , Al‐Majali, A. , & Tibbo, M. (2017). Role of small ruminants in the epidemiology of foot‐and‐mouth disease in Sudan. Bulletin of Animal Health and Production in Africa, 65, 145–156.

[tbed14472-bib-0028] Roeder, P. L. , & Le Blanc Smith, P. M. (1987). Detection and typing of foot‐and‐mouth disease virus by enzyme‐linked immunosorbent assay: A sensitive, rapid and reliable technique for primary diagnosis. Research in Veterinary Science, 43, 225–232. 10.1016/S0034-5288(18)30778-1 2825310

[tbed14472-bib-0029] Rweyemamu, M. , Roeder, P. , Mackay, D. , Sumption, K. , Brownlie, J. , Leforban, Y. , Valarcher, J. F. , Knowles, N. J. , & Saraiva, V. (2008). Epidemiological patterns of foot‐and‐mouth disease worldwide. Transboundary and Emerging Diseases, 55, 57–72. 10.1111/j.1865-1682.2007.01013.x 18397509

[tbed14472-bib-0030] Samuel, A. R. , & Knowles, N. J. (2001). Foot‐and‐mouth disease type O viruses exhibit genetically and geographically distinct evolutionary lineages (topotypes). Journal of General Virology, 82, 609–621. 10.1099/0022-1317-82-3-609 11172103

[tbed14472-bib-0031] Shaw, A. E. , Reid, S. M. , Ebert, K. , Hutchings, G. H. , Ferris, N. P. , & King, D. P. (2007). Implementation of a one‐step real‐time RT‐PCR protocol for diagnosis of foot‐and‐mouth disease. Journal of Virological Methods, 143, 81–85. 10.1016/j.jviromet.2007.02.009 17397937

[tbed14472-bib-0032] Snowdon, W. A. (1966). Growth of foot‐and mouth disease virus in monolayer cultures of calf thyroid cells. Nature, 210(5040), 1079–1080. 10.1038/2101079a0 4288087

[tbed14472-bib-0033] Thompson, J. D. , Higgins, D. G. , & Gibson, T. J. (1994). CLUSTAL W: Improving the sensitivity of progressive multiple sequence alignment through sequence weighting, position‐specific gap penalties and weight matrix choice. Nucleic Acids Research, 22, 4673–4680. 10.1093/nar/22.22.4673 7984417 PMC308517

[tbed14472-bib-0034] Ularamu, H. G. , Ibu, J. O. , Wood, B. A. , Abenga, J. N. , Lazarus, D. D. , Wungak, Y. S. , Knowles, N. J. , Wadsworth, J. , Mioulet, V. , King, D. P. , Shamaki, D. , & Adah, M. I. (2017). Characterization of foot‐and‐mouth disease viruses collected in Nigeria between 2007 and 2014: Evidence for epidemiological links between West and East Africa. Transboundary and Emerging Diseases, 63, 1–10. 10.1111/tbed.12584 27718336

